# The impact of probiotic administration *in vivo* on peritoneal mouse macrophages infected by *Leishmania amazonensis ex vivo*

**DOI:** 10.1590/0074-02760250014

**Published:** 2025-09-22

**Authors:** Lauren Van den Broeck, Raquel Silva de Azevedo, Ludmila Ferreira de Almeida Fiuza, Marcos Meuser Batista, Cynthia Machado Cascabulho, Ewout Van de Velde, Serge Van Calenbergh, Guy Caljon, Maria de Nazaré Correia Soeiro

**Affiliations:** 1 Fundação Oswaldo Cruz-Fiocruz, Instituto Oswaldo Cruz, Laboratório de Biologia Celular, Rio de Janeiro, RJ, Brasil; 2 University of Antwerp, Laboratory of Microbiology, Parasitology and Hygiene, Wilrijk, Belgium; 3 Fundação Oswaldo Cruz-Fiocruz, Instituto Oswaldo Cruz, Plataforma de Citometria, Rio de Janeiro, RJ, Brasil; 4 Laboratory of Medicinal Chemistry, Belgium

**Keywords:** leishmaniasis, Leishmania (L.) amazonensis, probiotics, peritoneal mouse macrophage, ex vivo

## Abstract

**BACKGROUND:**

The microbiome is fundamental in the host’s immunobiology and dysbiosis leads to pathological conditions, potentially affecting parasitic diseases.

**OBJECTIVES:**

To investigate how oral probiotics affect infection and antiparasitic treatment of *Leishmania* in macrophages.

**METHODS:**

Swiss mice were orally treated with 10^9^ colony forming units (CFU) multi- or single-strain probiotic formulations (PB8, Bifilac), their peritoneal mouse macrophages (PMMs) were obtained and infected *ex vivo* with *L. amazonensis* amastigotes. The effects of prior probiotic administration on *ex vivo* infection and treatment responses to 1 µM miltefosine and *N* ^6^-methyltubercidin were evaluated. Flow cytometry measured the inflammatory mediator release in the supernatant of the PMMs.

**FINDINGS AND MAIN CONCLUSIONS:**

PB8 or Bifilac administration significantly reduced (p < 0.05) *ex vivo* infection of PMMs from male mice by 27% and 12%, respectively. No gender-dependent effect of probiotics was observed. No improved antiparasitic activity of 1 µM miltefosine or *N* ^6^-methyltubercidin was observed in probiotic-treated PMMs. *Ex vivo Leishmania* infection stimulated tumour necrosis factor (TNF), MCP-1, and interleukin-6 (IL-6) production by PMMs (p *<* 0.05). A trend of increase was recorded with elevated levels of TNF and IL-6 in PB8-treated male groups (around 43 and 52%, respectively) but were not statistically significant. Collectively, probiotic treatment of mice influences *Leishmania* infection in PMMs. Clinical applications in leishmaniasis warrant further studies.

Leishmaniasis belongs to the group of neglected tropical diseases (NTDs), with an estimated 1 billion people at risk of infection, mostly in tropical and subtropical regions.^1^ Leishmaniasis is caused by a protozoan parasite of the genus *Leishmania* and is transmitted through the bite of infected sand flies. When an infected sand fly takes a blood meal, the metacyclic promastigotes will be inoculated into the mammalian host. They can then infect, differentiate, and replicate as amastigotes inside parasitophorous vacuoles of professional phagocytic cells.^2^ Although the parasites can infect other cell types such as fibroblasts, their main hosts are the mononuclear phagocytes.^3^ ^,^ ^4^ ^,^ ^5^ ^,^ ^6^

This intracellular protozoan can establish diseases of varying severity, ranging from self-healing skin ulcers to visceral forms, affecting internal organs and potentially being fatal if left untreated.^1^ ^,^ ^2^ ^,^ ^7^ ^,^ ^8^ Leishmaniasis is associated with impoverished populations and regions with poor living conditions, limited access to healthcare, and inadequate housing.^1^ ^,^ ^2^ The healthcare costs and the loss of productivity have major economic consequences, especially in vulnerable communities. Effective treatment is difficult due to many factors, including the great diversity of parasite and host genetics, complex life cycle, co-infections and re-infections in endemic areas, comorbidities, nutritional stress, among others.^2^ ^,^ ^9^ Drug resistance represents a special limiting factor for successful treatment and has been largely reported in several continents.^10^ ^,^ ^11^ ^,^ ^12^ Currently, there are no human vaccines on the market.^1^ ^,^ ^8^

*Leishmania amazonensis* is a New World, zoonotic *Leishmania* species belonging to the *L. mexicana* complex,^13^ that represents an important public health problem in South America where it has been associated with all three main clinical forms of leishmaniasis in humans: cutaneous, mucocutaneous, and visceral.^14^ Most frequently, infection with *L. amazonensis* causes one or more ulcerating lesions.^8^ Cutaneous leishmaniasis (CL) is usually self-limiting, but transversion to diffuse cutaneous leishmaniasis or mucocutaneous leishmaniasis can occur, depending on various factors, including the *Leishmania* spp., immunological status, and genetic predisposition of the host.^15^ ^,^ ^16^ However, self-healing can take months or even years and can leave atrophic scarring of the skin, in addition to the occurrence of secondary opportunistic fungal and bacterial infections that worsen the disease outcome. Treatment can accelerate the healing process and decrease the risk of mucosal dissemination, relapse, severe morbidity, and mortality.

Currently used drugs to treat CL comprise pentavalent antimonial (Sb^v^) compounds [meglumine antimoniate (MA) and sodium stibogluconate (SSG, Pentostam^®^)], pentamidine, amphotericin B deoxycholate (AmB-D) or it’s liposomal form (L-AmB-AmBisome^®^), miltefosine (Milteforan™), and paromomycin.^1^ ^,^ ^2^ ^,^ ^12^ ^,^ ^17^ However, their use is often costly and causes frequent adverse effect, like cardiotoxicity, nephrotoxicity and gastrointestinal alterations. The emergence of resistance against conventional drugs,^16^ ^,^ ^18^ ^,^ ^19^ frequent side effects, troubling co-infections with human immunodeficiency virus (HIV) and the limited funding for the development of novel drugs compels researchers and global health agencies to explore alternative strategies for combating and managing this significant neglected disease.

Microbiota play several roles in the metabolism of vertebrate and invertebrate hosts. The interplay between the nature and number of microorganisms that reside within and on the host influences its physiology and metabolism, and dysbiosis can lead to several pathological conditions including increased vulnerability to parasitic infections.^20^ ^,^ ^21^

This study investigates the potential use of probiotics as a supportive treatment and as a novel strategy for managing leishmaniasis. According to the definition of the World Health Organization (WHO),^22^ probiotics are: “live microorganisms that, when administered in adequate amounts, confer a beneficial health effect on the host”.^23^ Presently, oral probiotics are not commonly indicated as therapeutic or adjuvant agents for skin conditions like CL.^21^ However, recognising that skin disorders frequently exhibit gut dysbiosis, the close interaction between the gut and skin may present valuable therapeutic opportunities in the field of dermatology. According to previous studies, oral probiotics cause immunomodulation through the gut-associated lymphoid tissue (GALT) components and Peyer’s patches, known as the primary inductive sites for the mucosal immune response, present in the gastrointestinal tract.^24^ ^,^ ^25^ ^,^ ^26^ The beneficial effects of oral probiotic administration on the progression of dermatoses, mainly atopic dermatitis,^27^ acne vulgaris^28^ ^,^ ^29^ ^,^ ^30^ and psoriasis^31^ ^,^ ^32^ have been demonstrated.^33^ ^,^ ^34^ ^,^ ^35^ ^,^ ^36^ The use of probiotics has also been tested in several experimental non-enteric parasitic infections (Table I) including *Plasmodium* spp.,^37^ ^,^ ^38^ *Babesia microti*,^39^ ^,^ ^40^ *Trypanosoma cruzi* ^41^ and *T. b. brucei*.^42^ As a result of the administration of probiotics in these infection models, the parasitic load decreased significantly, and a more effective immune response was generated.

**TABLE I:** Effect of probiotics on non-intestinal parasitic infections in animal models. Adapted from Mukhopadhyay & Ganguly^43^

Parasitic pathogen	Probiotics studied	Host	Treatment	Efficacy	Reference
*Plasmodium chabaudi*	*Lactobacillus casei* ATCC7469	Mouse	7 - 15 days before infection	25% - 50% reduction	^37^
*Babesia microti*	*L. casei* ATCC7469	Mouse	0 - 7 days before infection	75% - 100% reduction	^40^
*Trypanosoma cruzi*	*L. casei* ATCC7469	Mouse	3-7 days before infection	75% - 100% reduction	^31^
*Trypanosoma brucei brucei*	*Saccharomyces cerevisiae*	Rat	28 days before infection	Significantly lower	^42^

Several studies^44^ ^,^ ^45^ ^,^ ^46^ using *L. major*-infected mice showed that the gut microbiome significantly influences the lesion’s self-healing capacity, independent of the adaptive immune system’s Th1 response. According to a study published in 2021 by Lopes et al.,^44^ the host gut microbiome is crucial for activating macrophages and promoting a resistance phenotype in mice infected with *L. major*.

With this study, we want to investigate the influence of pretreatment of male and female Swiss mice with probiotics on the *ex vivo* infection of collected peritoneal macrophages by *L. amazonensis*.

## MATERIALS AND METHODS

*Animals* - Adult male and female BALB/c and Swiss mice (18-20 g) were obtained from the animal facilities of Institute of Science and Biomodels Technology/Fiocruz/RJ/Brazil (ICTB). All mice were housed five animals per cage, kept in a room at 20 to 24ºC under a 12-hour light-dark cycle, and provided with sterile water and chow *ad libitum*. All mice underwent an acclimatisation period of seven days before starting the experiments.

*Parasites* - *Leishmania amazonensis* (MHOM/BR/77/LTB0016) were used throughout the study and were maintained as amastigotes by serial passages into footpads (subcutaneously) of male BALB/c mice every four-six weeks. BALB/c mice were inoculated with 20 µL containing 10^6^ amastigotes. Thirty days post-infection, the skin lesions were removed aseptically and mechanically dispersed by pipetting, and the amastigotes purified as reported.^47^

*Probiotics* - PB8 probiotics (composed of *Lactobacillus acidophilus* LA-14^®^, *Bifidobacterium lactis* BL-04^®^, *Lactobacillus salivarius* LS-33^®^, *Bifidobacterium bifidum* BB-06™, *Bifidobacterium longum* BL-05™, *Lactobacillus rhamnosus* LR-32^®^, and *Lactobacillus casei* LC-11^®^ elements) were purchased from Nutrition Now. *Lactobacillus rhamnosus* was purchased as Bifilac (Geflora). Administration of probiotics to mice was done by oral gavage (18 G needle). To obtain the correct dosage, the contents of the probiotic capsules were dissolved in distilled and sterile water (one capsule diluted in 1.4 mL) and 0.2 mL given per mouse.

*Chemicals* - Brewer thioglycolate was obtained from Sigma-Aldrich (B2551-500G) and prepared by dissolving 3 g of sodium thioglycolate in 100 mL of distilled water. *N* ^6^-methyltubercidin was synthesised as reported.^48^ A stock solution of *N* ^6^-methyltubercidin (30 mM) was prepared in dimethyl sulfoxide (DMSO). The final in-test concentration of DMSO was below 0.6% to prevent non-specific toxicity to the host cells.^49^ A stock solution (30 mM) of miltefosine (Sigma-Aldrich) was prepared in ultrapure and sterile water.^47^

*Mice treatment* - Swiss male and female mice (n = 3-5 per group) received 10^9^ colony forming units (CFU) of either PB8 probiotic solution or *L. rhamnosus* (Bifilac) for five consecutive days via oral gavage (18 G) before the obtention of peritoneal mouse macrophages (PMMs). As a control, the vehicle group (n = 3-5) received only distilled, sterile water in the same quantities and for the same duration. Four days prior to the isolation of the PMMs, each group received intraperitoneal administration (26 G) of 1 mL Brewer thioglycolate medium to stimulate migration of macrophages to the peritoneal cavity. 24 h after the last probiotic or vehicle administration, the mice were euthanised, and PMM were collected and processed as described in ‘*ex vivo* assays’.

*Ex vivo assays* - The PMM culture was obtained from Swiss male or female mice (18-20 g) previously stimulated with 3% thioglycolate medium (i.p.). PMMs were collected by injecting the mouse peritoneum with 10 mL of cold RPMI 1640 medium with phenol red. After massaging the fluid-filled abdomen for 1 min to dislodge attached cells into the peritoneal fluid, the medium containing PMMs was extracted again and checked for any contamination by light microscopy. The medium and cells were kept on ice to avoid PMM adhesion on the tubes until quantification by light microscopy. Then the isolated PMMs were seeded in 24-well plates (3 × 10^5^ cells/well) and maintained at 37ºC and 5% CO_2_ in RPMI 1640 medium (pH 7.2 to 7.4) with phenol red, supplemented with 1% (v/v) L-glutamine 200 mM, 1% (v/v) penicillin-streptomycin (10,000 U/mL-10 mg/mL), and 10% (v/v) foetal bovine serum (FBS).^47^

*PMM infection by amastigotes of L. amazonensis* - After 24 h of plating, the PMM cultures were rinsed with phosphate buffered saline (PBS) and infected with amastigotes of *L. amazonensis* at 2.5:1 parasite:host cell ratio with an interaction time of 2 h, as reported.^50^ To favour the parasite-host cell interactions to occur, 500 µL of medium containing parasites was added to each well of the 24-well plates instead of the usual 1 mL volume of medium. The plates were then incubated at 37ºC and 5% CO_2_. After the interaction time, the medium containing non-internalised parasites was removed, the host cells were rinsed twice with PBS, and the medium replaced with supplemented RPMI 1640 medium. After 24 h and 48 h of incubation, the cultures were fixed using Bouin solution and stained using 10% Giemsa (Sigma-Aldrich).^50^ The coverslips were then fixed on a microscopic slide with Permount™ mounting medium. At least 200 randomly selected cells per microscopic slide were examined at ×1000 magnification under a Zeiss Axioplan microscope (Carl Zeiss Inc., Thornwood, NJ) to determine the percentage of PMMs containing amastigotes, the number of amastigotes per infected host cell and the infection indexes (multiplication of the former two parameters - the percentage of infected host cells by the number of parasites per infected host cell).

**Fig. 1:**
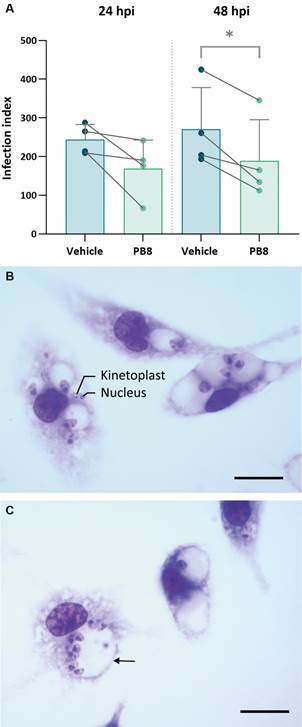
(A) infection indexes of peritoneal mouse macrophages (PMMs) obtained from male Swiss mice previously treated with PB8 or vehicle, and *ex vivo* infected with *Leishmania amazonensis* amastigotes, at 24 h and 48 h post infection (hpi). The individual mean data of four independent experiments, performed in technical duplicates, are shown in the bar chart. The connecting lines illustrate the reduction of the infection index due to the application of probiotics in independent experiments. Each bar represents the average infection index of the four independent assays. The asterisk indicates that differences are statistically significant (p < 0.05). (B - C) Light microscopy images of Giemsa-stained, *L. amazonensis*-infected PMMs (2.5:1 parasite:host cell ratio) from male mice treated with vehicle (B) and PB8 (C) at 48 h p.i.. PMMs display intracellular amastigotes with their characteristic darker-stained nucleus and kinetoplast in large, communal parasitophorous vacuoles (arrow). (Bar = 10 µm).

*Drug activity: ex vivo leishmanicidal analysis* - The phenotypic screenings were performed by infecting PMM cultures as above described. Following the 2 h incubation time for parasite internalisation, cultures were rinsed twice with PBS and the medium replaced with supplemented RPMI 1640 medium or medium containing 1 µM miltefosine (a reference drug for leishmaniasis) or *N* ^6^-methyltubercidin for 24 and 48 h. Subsequently, the cultures were rinsed with PBS, fixed for 5 min with Bouin solution, and stained for 15 min with Giemsa solution 10% (Sigma-Aldrich) as above described. Analogously, the samples will be subsequently evaluated by light microscopy and microphotographs made using the Zeiss AxioObserver MI microscope (Oberkochen, Germany). The percentage of infected host cells, the number of parasites per infected cell, and the corresponding infection indexes were scored by direct light microscopy quantification, as reported.^50^ ^,^ ^51^ ^,^ ^52^ The results are expressed as a percentage of reduction in parasite burden (infection indexes). At least 200 cells per microscopic slide were analysed. Only parasites with well-defined nuclei and kinetoplasts (Fig. 1) were counted as surviving, since irregular structures could mean parasites undergoing death.

*Cytometric bead array* - Cytometric bead array (CBA) Mouse Inflammation Kits were purchased from BD Biosciences (USA) and used per manufacturer’s instructions. This kit was used to quantitatively measure the interleukin-6 (IL-6), interleukin-10 (IL-10), monocyte chemoattractant protein-1 (MCP-1), interferon- (IFN-), tumour necrosis factor (TNF) and interleukin-12p70 (IL-12p70) protein levels released in the supernatant of PMM cultures from mice previously treated with PB8 or vehicle, as described above. Two independent assays were performed in duplicate. The cultures were then infected or not with *L. amazonensis* (MHOM/BR/77/LTB0016) and treated or not with 1 µM of miltefosine or *N* ^6^-methyltubercidin. The supernatant from each PMM culture was collected 48 h post infection and stored at -80ºC. A serial dilution of Mouse inflammation standards was used to construct a standard curve for the quantitative analysis of the test samples. The mouse inflammation capture beads were mixed and 25 µL of this mixture was added to 25 µL of the samples or mouse inflammation standards in a 96-well plate. Then 25 µL of detection reagent is added to each well and the plate incubated at room temperature, protected from light, for two hours. Then 100 µL of wash buffer is added to each well and the plate is centrifuged at 200×*g* for 5 min. The supernatant containing any molecules not attached to the beads, such as unbound detection antibodies, is removed and the pellet is resuspended in 100 µL of wash buffer. All samples were then analysed by the Cytoflex S (Beckman Coulter) flow cytometer. The generated data was analysed using the FCAP Array (BD Biosciences) software. The CBA analysis using flow cytometry was implemented in this study due to its high sensitivity and ability to multiplex multiple cytokines simultaneously.^53^

*Statistical analysis* - Statistical analysis was conducted in GraphPad Prism 10.4.1 (627) by paired *t*-test or one-way analysis of variance (ANOVA) with a significance level of p ≤ 0.05 [95% confidence interval (CI)].

*Ethical statement* - All procedures were carried out under the biosafety guidelines of the Oswaldo Cruz Institute (IOC/Fiocruz), and all animal procedures complied with the guidelines established by the FIOCRUZ Committee of Ethics for the Use of Animals, resolution (CEUA L-017/2023).

## RESULTS

*Effect of probiotic administration in vivo upon PMM infection ex vivo by L. amazonensis* - Male Swiss mice (n = 3-5) were treated for five consecutive days with 10^9^ CFU of a multi-strain probiotic (PB8), then stimulated with 3% thioglycolate before PMM collection. The PMMs were seeded and then infected for 2 h with *L. amazonensis* amastigotes. After 24 and 48 h, the cultures were fixed and stained with 10% Giemsa. The parasite burden of the infected PMMs obtained from the different groups of mice was determined by phenotypic analysis through light microscopy counting the percentage of infected cells and the number of amastigotes per infected cell to determine the infection indexes.

Our results demonstrate that the infection indexes of PMMs collected from PB8 mice (of four independent assays) were consistently lower (p < 0.05) compared to vehicle groups. As decreases were observed at both timepoints, but more consistently at 48 h post infection (p.i.), we only considered the 48 h timepoint for further studies (Fig. 1).

**Fig. 2:**
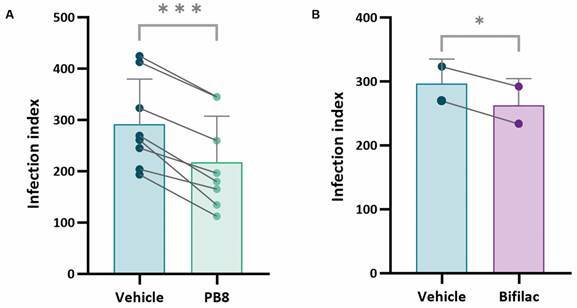
infection indexes of peritoneal mouse macrophages (PMMs), obtained from male Swiss mice treated with (A) PB8, (B) Bifilac or (A and B) vehicle, 48 hpi with *Leishmania amazonensis* amastigotes. Each data point represents the average infection index of two duplicate samples within one independent assay. The connecting lines indicate the reduction of the infection index due to the application of probiotics in independent experiments. The ^*^ and ^***^ indicate statistically significant differences of p ≤ 0.05 and p ≤ 0.0005, respectively.

Next, further assessment was performed using a single strain - *L. rhamnosus* (Bifilac), which was run in parallel to PB8 assays (Table II, Fig. 2). As noticed with PB8, Bifilac reduced the percentage of infected PMMs, the number of parasites per cell and consequently the respective infection indexes (Fig. 2). The effect of PB8 compiled from eight independent assays using male Swiss mice, resulted in a significant (p < 0.05) reduction in all measured parameters compared to the PMMs from the vehicle-treated mice (Fig. 2). Notably, the infection index decreased by approximately 27%. Bifilac, evaluated in two independent assays in technical duplicates, significantly reduced the infection index by approximately 12% (p < 0.05), while no significant effects were observed for the other measured parameters. Although not statistically significant (p > 0.05), PB8 was 2.4-fold more effective than Bifilac in reducing the infection index in PMM collected from male Swiss mice (Table III).

**TABLE II:** Percentage of reduction of the number of infected cells, the number of amastigotes per infected cell and the infection indexes of *Leishmania amazonensis*-infected peritoneal mouse macrophages (PMMs), 48 h post infection (hpi), after *in vivo* PB8 or Bifilac treatment. Since the PB8 results met normality criteria in multiple tests, paired t-tests were used for the statistical analysis of both PB8 and Bifilac data

Average values of percentage infection reduction of *L. amazonensis*-infected PMMs: probiotics x vehicle	PB8^a^	Bifilac^b^
Percentage infected cells	14.3 (p = 0.0005)	6.2 (p = 0.2143)
No. of amastigotes per infected cell	15.3 (p = 0.0055)	5.8 (p = 0.3083)
Infection index	27.2 (p = 0.0001)	11.5 (p = 0.0415)

*a*: the percentage reduction of the infection parameters of the PMMs obtained from PB8-treated mice compared to vehicle-treated mice, is the average percentage reduction of eight independent assays; *b*: the percentage reduction of the infection parameters of the PMMs obtained from Bifilac-treated mice compared to vehicle-treated mice, is the average percentage reduction of two independent assays.

**TABLE III:** The percentage reduction of the infection indexes of peritoneal mouse macrophages (PMMs) at 48 h post infection (hpi) due to *in vivo* PB8 or Bifilac administration in male and female Swiss mice in comparison to vehicle-treated counterparts

Average percentage (%) reduction of infection indexes after *in vivo* probiotic administration		Probiotic	
		PB8	Bifilac
Mouse gender	Male	22.3 (p = 0.0040)^a^	11.5 (p = 0.0415)^b^
	Female	25.4 (p = 0.1264)^a^	28.7 (p = 0.1939)^b^

*a*: the data included the results of four independent assays run in parallel; *b*: the data included the results of two independent assays run in parallel.

*Effect of probiotic administration in vivo upon PMM infection ex vivo by L. amazonensis: comparison among animal genders* - Next, aiming to determine a possible gender-dependent effect to probiotic treatment, both male and female Swiss mice (n = 3-5) were treated for five consecutive days with 10^9^ CFU PB8 and Bifilac and then stimulated with 3% thioglycolate before PMM obtention. PMMs were infected for 2 h with *L. amazonensis* amastigotes. After 48 h, the cultures were fixed, stained and the number of infected PMMs, amastigotes per cell and corresponding infection indexes determined with light microscopy. Four independent assays in duplicate were performed comparing PB8 and vehicle and two independent assays in duplicate comparing Bifilac and vehicle (sterile water).

**Fig. 3:**
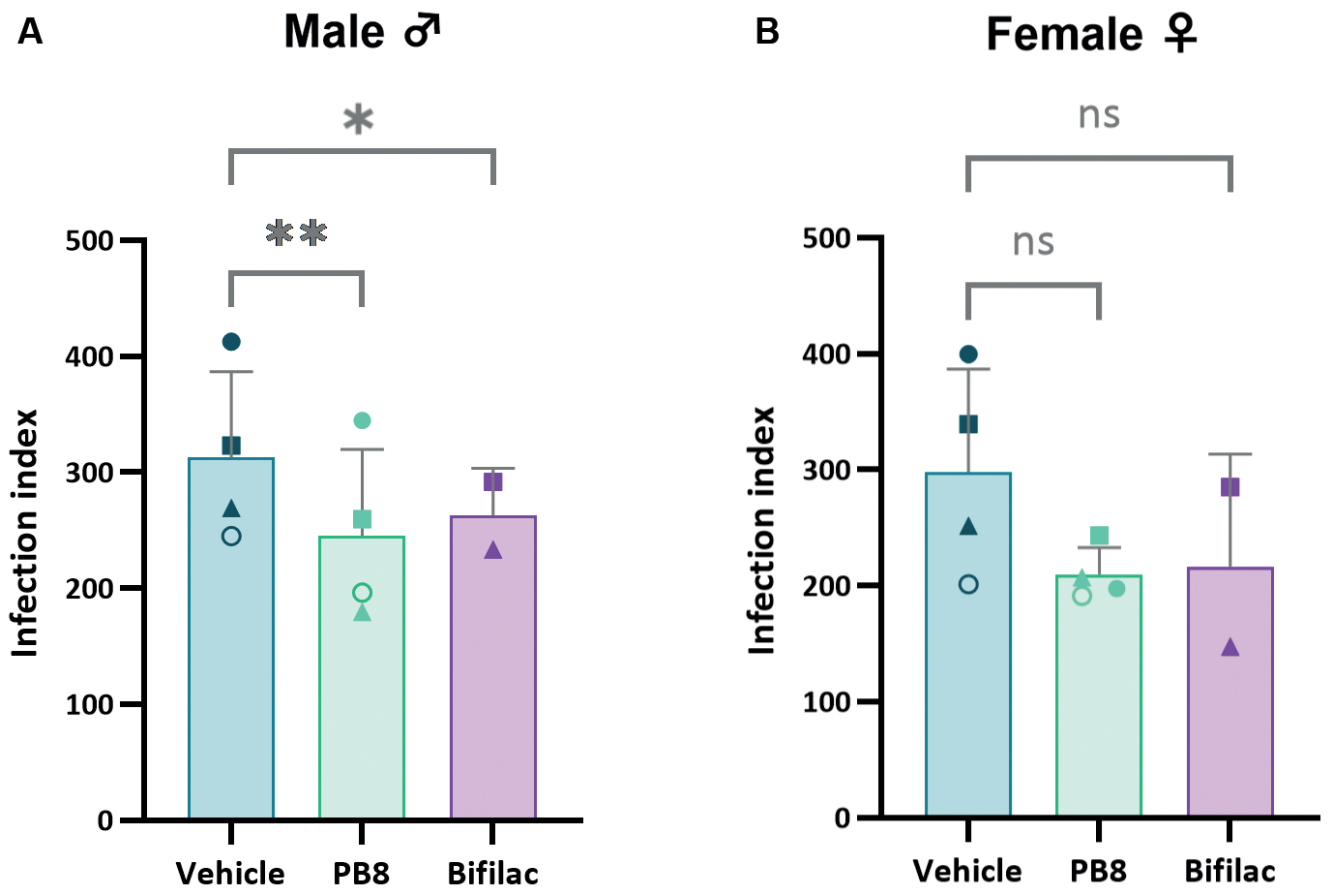
infection indexes of *Leishmania amazonensis*-infected peritoneal mouse macrophages (PMMs) obtained from male (A) and female (B) Swiss mice treated with PB8, Bifilac or vehicle. Four and two independent assays were performed in technical duplicate for PB8 and Bifilac, respectively. Each data point shape represents an independent assay. Statistical significance is presented as ‘ns’ (p > 0.05), ^*^(p ≤ 0.05) or ^**^(*p* ≤ 0.005).

In the vehicle-treated groups, the infection indexes of PMMs derived from male and female mice were comparable, measuring 312 ± 79 and 298 ± 88, respectively. Again, regardless of sex, parasitism was consistently higher in PMMs from vehicle-treated animals compared to those receiving PB8 or Bifilac (Fig. 3)- PB8 administration resulted in an average reduction of the infection index by 22% in male mice and 25% in female mice, with no statistically significant difference between the sexes (p > 0.9999) (Table III). Bifilac exhibited a 2.5-fold greater reduction of the infection index in PMMs from female mice compared to male mice, although this difference was not statistically significant. Infection index reductions following PB8 and Bifilac treatment were statistically significant in male mice (p = 0.0040) but not in female mice (p > 0.05), likely due to greater variability across independent assays.

**Fig. 4:**
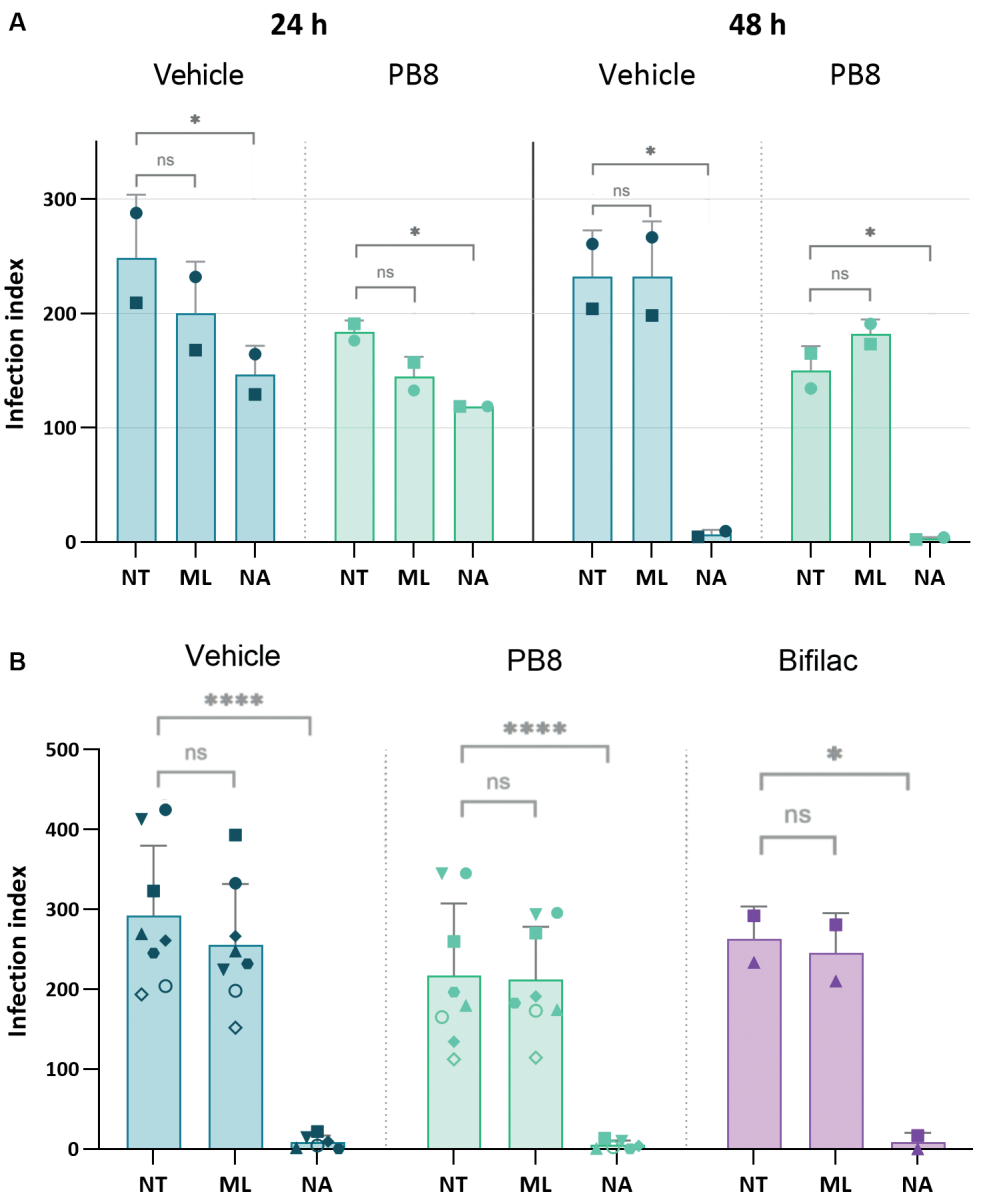
(A) infection indexes of peritoneal mouse macrophages (PMMs) obtained from PB8 and vehicle-treated male Swiss mice, infected with *Leishmania amazonensis* amastigotes and then treated for 24 h and 48 h with 1 µM of miltefosine (ML) or nucleoside analogue *N* ^6^-methyltubercidin (NA), in comparison to untreated PMMs (NT). (B) Comparison infection indexes following *in vivo* probiotic (PB8 and Bifilac) administration and *ex vivo* treatment for 48 h with 1 µM of ML or NA. Each data point shape represents an independent assay. Statistical significance is presented as ‘ns’ (p > 0.05), ^*^(*p* ≤ 0.05) and ^****^(p ≤ 0.0001).

*Effect of probiotic administration in vivo upon PMM infection ex vivo by L. amazonensis: analysis on drug efficacy* - To determine whether the probiotics influence the effectiveness of drug treatment, male and female Swiss (n = 3-5) mice received orally 10^9^ CFU of PB8 and Bifilac or sterile water for five consecutive days and were stimulated with 3% thioglycolate. The PMMs were infected with *L. amazonensis* amastigotes and then treated (for 24 and 48 h) with a single concentration (1 µM) of miltefosine and *N* ^6^-methyltubercidin (Fig. 4, Table IV). PMMs were fixed, stained and the number of infected host cells, amastigotes/per cell and corresponding infection indexes determined by light microscopy.

**TABLE IV:** Average percentages of infection variation in peritoneal mouse macrophages (PMMs) obtained from probiotic- or vehicle-treated male Swiss mice, infected with *Leishmania amazonensis* amastigotes and treated for 24 h and 48 h with 1 µM miltefosine or nucleoside analogue *N* ^6^-methyltubercidin. P-values are calculated comparing the drug-treated and untreated PMMs within each probiotic group

Average percentage (%) variation of infection index (treated x untreated samples)	Miltefosine		N^6^-methyltubercidin	
	24 h	48 h	24 h	48 h
Vehicle	-17.21 (p = 0.0201)^a^	-10.19 (p = 0.2254) ^b^	-40.62 (p = 0.1332)^c^	-97.25 (p = 0.0002)^d^
PB8	-1.67 (p = 0.3475)^a^	1.74 (p = 0.6807)^b^	-35.25 (p = 0.0713)^c^	-97.85 (p = 0.0009)^d^
Bifilac		-6.90 (p = 0.2176)^c^		-97.07 (p = 0.0520)^c^

*a*: the data included the results of four independent assays run in parallel; *b*: the data included the results of eight independent assays run in parallel; *c*: the data included the results of two independent assays run in parallel; *d*: the data included the results of six independent assays run in parallel.

**Fig. 5:**
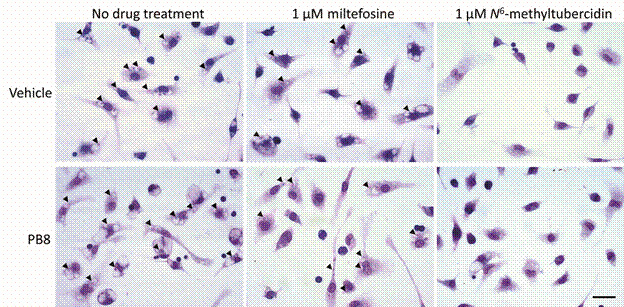
light microscopy images of Giemsa-stained peritoneal mouse macrophages (PMMs) obtained from PB8 and vehicle-treated male Swiss mice infected *ex vivo* with amastigotes of *Leishmania amazonensis* and treated or not for 48 h with 1 µM miltefosine or nucleoside analogue, *N* ^6^-methyltubercidin. The arrows indicate the intracellular amastigotes. The scale bar represents 20 μm.

The vehicle-treated group gave higher infection indexes than PB8- or Bifilac-treated mice across all drug treatments for both genders of mice (Fig. 4). While miltefosine did not give a considerable reduction in both timepoints (at 1 µM), *N* ^6^-methyltubercidin was notably more potent after 48 h as compared to 24 h (p < 0.0340). *N* ^6^-methyltubercidin was 61-fold (p = 0.0001) and 71-fold (p < 0.0001) more active than miltefosine in vehicle- and PB8-treated mice, respectively. After 48 h, 1 µM of *N* ^6^-methyltubercidin resulted in an almost complete sterilisation of the PMMs, reaching > 97% of parasitism decline (Figs 4-5, Table IV).

When compared to vehicle-treated animals, probiotics had no statistically significant effect on the potency of miltefosine or nucleoside analogue *N* ^6^-methyltubercidin (p > 0.05) (Table IV).

The same assays were performed for PMMs obtained from female mice. There was no significant (p > 0,05) gender-dependent effect of *in vivo* probiotic administration on drug efficacy *ex vivo* at 1 µM of the studied drugs.

*Effect of probiotic administration in vivo on the release of inflammatory mediators by PMM during infection ex vivo by L. amazonensis* - The potential mode of action of the probiotics was investigated. Male and female Swiss mice (n = 3-5) were treated for five consecutive days with 10^9^ CFU of PB8 and stimulated with 3% thioglycolate before PMM obtention. The PMMs were infected for 2 h with *L. amazonensis* amastigotes, after 48 h, their supernatant collected and analysed by flow cytometry to measure different inflammatory mediators such as: interleukin 12p70 (IL-12p70), TNF, interferon gamma (IFN-y), monocyte chemoattractant protein 1 (MCP-1), IL-10 and IL-6.

**Fig. 6:**
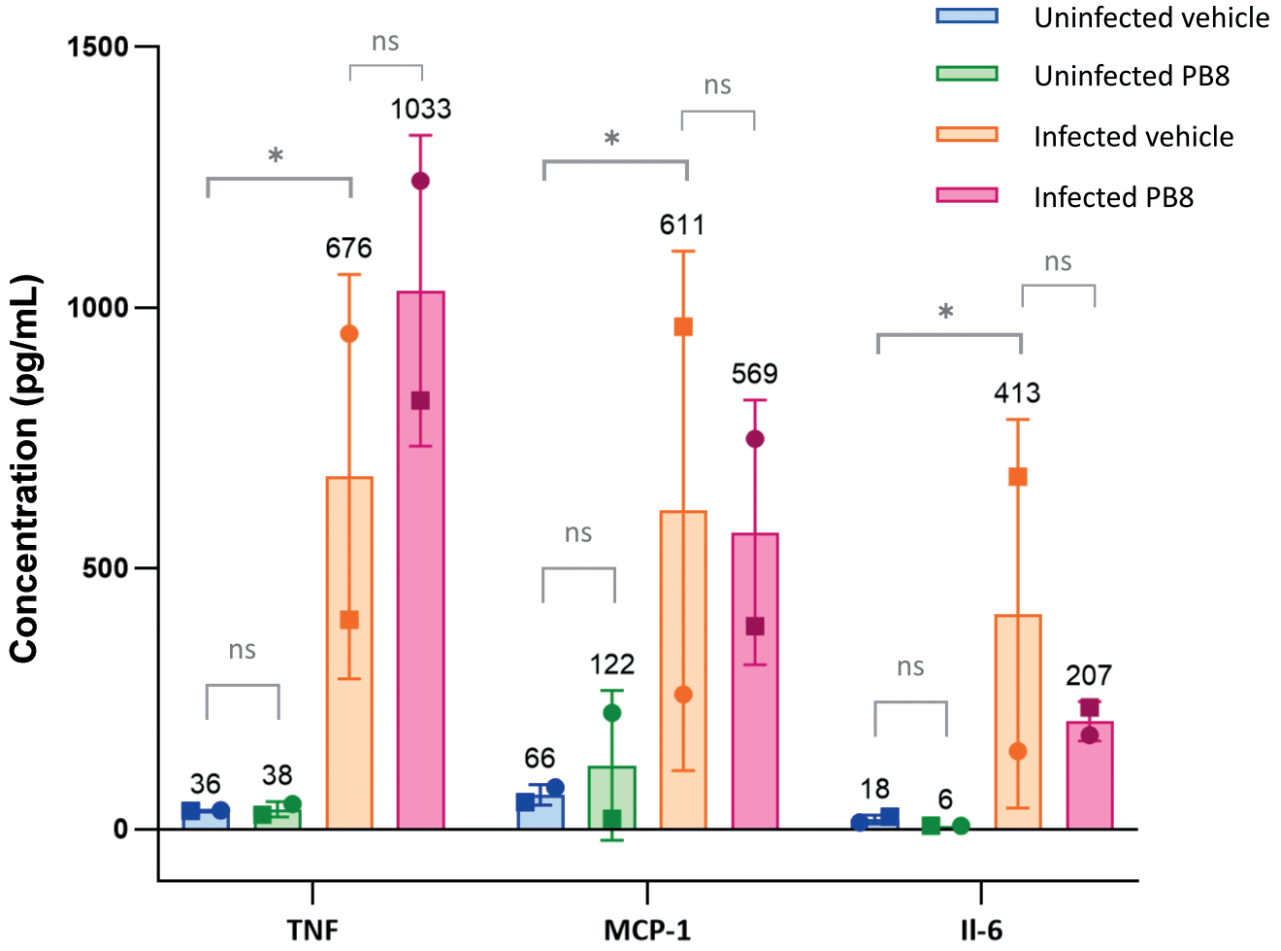
concentration of inflammatory mediators TNF, MCP-1 and IL-6 in the supernatant of *Leishmania amazonensis*-infected peritoneal mouse macrophages (PMMs), obtained from probiotic (PB8) or vehicle treated mice (n = 3-5), measured by flow cytometry using a Cytometric Bead Array (CBA) kit. The bars and error bars represent the mean and standard deviation of two independent assays in duplicate. Each data point shape represents an independent assay. Statistical significance is presented as ‘ns’ (p > 0.05) and ^*^(p ≤ 0.05).

In all groups (vehicle and PB8) there were statistically significant increases in TNF, MCP-1 and IL-6 levels in infected cultures as compared to uninfected samples (p < 0.05) (Fig. 6). Although TNF and IL-6 levels were higher in PB8-groups compared to their respective vehicle-treated mice, no statistical significances were found (p > 0.05) (Fig. 6). IL-12, IFN- and IL-10 could not be accurately analysed because of measurements below the limit of detection (concentrations = 0 pg/mL).

## DISCUSSION

The lack of a human vaccine or chemoprophylaxis for CL underscores the importance of reservoir population elimination and vector control to reduce the incidence of leishmaniasis. In addition, there is a constant search for novel treatment strategies to effectively treat CL infections with fewer adverse effects and lower costs.

Our studies indicate that *in vivo* probiotic administration influences the infection rate and parasite burden of *ex vivo* infected peritoneal macrophages. The results from male Swiss mice pretreated with either the multi-strain PB8 or the single-strain Bifilac (*L. rhamnosus*) demonstrated a significant impact on the *ex vivo* infection of PMMs with *L. amazonensis* amastigotes, with average infection index reductions of 27% (p = 0.0001) and 12% (p = 0.0415), respectively. PB8 administration resulted in a statistically significant reduction in both the percentage of infected cells (p = 0.0005) and in the number of amastigotes per infected cell (p = 0.0055). A comparable, yet not statistically significant effect was observed when investigating female Swiss mice, with reductions of approximately 25% and 29% in the infection index following *in vivo* administration of PB8 and Bifilac, respectively.

It has previously been established that multi-strain probiotic mixes have more beneficial effects and are more effective than individual bacterial strains.^54^ ^,^ ^55^ ^,^ ^56^ ^,^ ^57^ In our experimental model using male Swiss mice, PB8 (a multi-strain probiotic) was 2.4 times more effective than Bifilac (a single strain-probiotic of *L. rhamnosus*), however this difference was not statistically significant.

The decrease (p < 0.01) of the number of infected cells and the number of amastigotes per infected cell following probiotic administration indicates that the parasite infection as well as the intracellular survival is reduced. This is in line with a study by Lopes et al.,^44^ who reported that the absence of microbiota in germ-free, female Swiss mice resulted in a higher susceptibility of macrophages to *L. major* infection. This may relate to decreased cell activation and iNOS production by the infected macrophages. Several other studies^41^ ^,^ ^42^ ^,^ ^43^ using *L. major*-infected mice showed that the gut microbiota significantly influences the lesion’s self-healing capacity. Commensals may influence *Leishmania* infection and disease outcome since they may drive monocyte movement from the bone marrow to different systemic sites in the body,^44^ ^,^ ^58^ ^,^ ^59^ ^,^ ^60^ ^,^ ^61^ and may activate their microbicidal machinery, contributing to parasite burden control. This study could not formally discern whether the probiotics had a stronger influence on parasite entry or on the macrophages’ ability to kill the intracellular amastigotes. An analysis of the PMM infection soon after the interaction (*e.g.*, 2 h) with amastigotes could provide more details regarding the impact on parasite internalisation and/or proliferation.

Several studies with different *Leishmania* spp. reported that there is a gender-related risk for the development of leishmaniasis. For instance, male hamsters are more susceptible than females to infection with *L. panamensis* or *L. guyanensis*.^62^ While male DBA/2 mice are more susceptible to *L. mexicana*, females are more susceptible to *L. major*.^63^ Therefore, research highlights that specific parasite-host combinations can be subjected to a sex bias.^64^ In this sense, we also compared the effect of probiotics on the infection development in PMM collected from both female and male mice. Our findings demonstrate that probiotics exhibit similar patterns in both sexes, leading to decreases in PMM parasitism *ex vivo*. Thus, our studies did not reveal significant differences when comparing PMMs obtained from male or female mice following *in vivo* probiotic administration. In addition, when drug activity (miltefosine and *N* ^6^-methyltubercidin) was analysed at a single concentration (1 µM), we found that there was no additional effect in PB8, and Bifilac-treated groups (p > 0.05) in mice of both sexes. Future studies using different concentrations of the drugs are desirable to check and confirm this aspect.

Our present analysis corroborates previous studies that reported higher leishmanicidal effect of nucleoside analogues as compared to the reference drug for leishmaniasis (miltefosine) and that this class of compounds are very promising for a future drug candidate to treat this tropical neglected disease^65^ ^,^ ^48^ as well as for Chagas disease (CD).^53^ ^,^ ^66^ While miltefosine did not show a larger reduction of infection in a time-dependent manner, *N* ^6^-methyltubercidin was notably more potent after 48 h compared to 24 h of drug exposure. After 48 h, a concentration of 1 µM of *N* ^6^-methyltubercidin resulted in an almost complete sterilisation of the PMMs. The reason for the time-dependent action may be related to the mode of action of these nucleoside derivatives. As these parasites are auxotrophic for purines, the time-dependent effect presently found (higher potency at later timepoints) could be rationalised by the fact that the nucleoside analogue only starts exerting an antiparasitic effect after the stock of endogenous purine nucleosides previously taken up by the parasites is exhausted. Miltefosine, on the contrary, has a direct mechanism of action associated with disturbing the lipid metabolism, membrane composition and mitochondrial activity of the parasite, resulting in an apoptosis-like cell death.^67^

Another aspect that was explored was the correlation of probiotic treatment with pro- and anti-inflammatory cytokine levels produced by PMMs. TNF, IL-6 and MCP-1 levels were increased in the supernatant of *L. amazonensis*-infected PMMs from all groups of mice (probiotic or vehicle) as compared to uninfected cultures. Our data corroborate in part the findings in human *L. amazonensis* infections that also report higher levels of TNF and IL-6.^68^ According to a study published in 2021 by Lopes et al.,^44^ the host gut microbiome is crucial for activating macrophages and promoting a resistance phenotype in mice infected with *L. major.* The number of infected macrophages increased and the number of iNOS+ macrophages diminished *in vivo* for dysbiotic and germ-free mice, compared to conventional mice. The absence of host microbiota in germ-free, female mice resulted in a lower production of both IL-12p70 and TNF upon stimulation of bone marrow-derived macrophages with LPS+IFN-γ. In the present study, the probiotics only mildly increased TNF and IL-6 released into the supernatant of PMMs from both male and female mice after treatment with PB8 but this was not statistically significant.

A previous study in 2019 tested the effect of *Lactobacillus bulgaricus* on macrophage activation to inhibit tumour growth. They concluded that the bacteria increased the pro-inflammatory activity of macrophages *in vitro, ex vivo* and *in vivo*.^69^ Another study^70^ reported RAW 264.7 macrophage activation to a pro-inflammatory state after probiotic administration with *L. acidophilus* and *Bacillus clausii*. In agreement with the findings of this investigation, Guha et al. found that treatment with *L. bulgaricus* increased the production of TNF and IL-6 as we also presently observed.

Previous analyses have shown the influence of some predominant components from the indigenous microbiota on systemic immunological responses during experimental CD in mice.^71^ In these experiments, germ-free mice were individually colonised with either Gram-positive (*Peptostreptococcus* sp.) and Gram-negative (*Bacteroides vulgatus*) obligate anaerobic bacteria or Gram-positive (*Enterococcus faecalis*) and Gram-negative (*E. coli*) facultative bacteria, representative of the prevalent human faecal microbiota, and then subjected to *T. cruzi* infection. The study found that gnotobiotic mice, mono-associated with *E. faecalis*, *B. vulgatu*s and *Peptostreptococcus*, had a significantly higher survival rate and a Th1 response (higher IFN-γ, TNF-α, and NO productions by spleen cell cultures), with increased serum levels of specific immunoglobulins displaying an IgG2a isotype, which is known to protect against *T. cruzi* and *Leishmania* infection. *T. cruzi* may manipulate the host’s gut microbiota, favouring its survival. For instance, a higher linoleic acid metabolism, associated with an important immunomodulating response involving T-cell recruitment and cytokine production, is observed in infected mice. Administration of probiotics could influence this process of host microbiota manipulation by *T. cruzi*. Probiotics also may compete with the parasites for nutrients and space and stimulate and prime the hosts’ immune system to react against infection.^72^

The goal of this study was to investigate the benefit of probiotics as a supportive co-treatment strategy for human leishmaniasis. Our findings demonstrate that probiotics can reduce experimental *ex vivo* infection of PMMs with *L. amazonensis*. Hence, adjuvant probiotics therapy may also be beneficial for the treatment of humans infected with *Leishmania*. Given the potential benefits of probiotics in bolstering the immune system, it is reasonable to consider their pre-emptive use and supportive add-on application during current treatments. However, findings from mouse models may not directly translate to humans due to species-specific differences in immune responses and microbiota composition. In our experimental mice models, animals receive daily probiotic administration (freshly prepared) through oral gavage. However, variations in dosage, frequency, and duration of probiotic treatment may differ from what occurs in human or natural settings, impacting the translational relevance of the findings. It is important to consider both the long-term and short-term effects of probiotic use. The relatively short duration of the study (presently using only five days of probiotic administration) may restrict the ability to observe the long-term effects of probiotic administration on PMM functions. Chronic exposure or repeated dosing may elicit different responses not captured in short-term experiments. Thus, although the direct translation of experimental models and clinical outcomes is not always correlated, preclinical studies as we presently report are the way to assess hypothesis, and the bulk of our data demonstrates the clear benefit of probiotic administration to provide better disease outcome for CL. Probiotic administration may act as an adjuvant therapy to be used along drugs, possibly under lower doses, resulting in less side effect and achieving better clinical outcomes.

While probiotics hold promise for combating parasitic infections and promoting overall health, their integration into public health strategies requires careful consideration. Therefore, further studies are needed to identify their beneficial use and to understand the microbiota’s influence on CL, paving the way for innovative approaches to disease management.
